# Soft Covering Based Rough Sets and Their Application

**DOI:** 10.1155/2014/970893

**Published:** 2014-09-09

**Authors:** Şaziye Yüksel, Zehra Güzel Ergül, Naime Tozlu

**Affiliations:** ^1^Department of Mathematics, Faculty of Science, Selçuk University, 42003 Konya, Turkey; ^2^Department of Mathematics, Faculty of Science and Art, Ahi Evran University, 40100 Kırşehir, Turkey; ^3^Department of Mathematics, Faculty of Science and Art, Niğde University, 51100 Niğde, Turkey

## Abstract

Soft rough sets which are a hybrid model combining rough sets with soft sets are defined by using soft rough approximation operators. Soft rough sets can be seen as a generalized rough set model based on soft sets. The present paper aims to combine the covering soft set with rough set, which gives rise to the new kind of soft rough sets. Based on the covering soft sets, we establish soft covering approximation space and soft covering rough approximation operators and present their basic properties. We show that a new type of the soft covering upper approximation operator is smaller than soft upper approximation operator. Also we present an example in medicine which aims to find the patients with high prostate cancer risk. Our data are 78 patients from Selçuk University Meram Medicine Faculty.

## 1. Introduction

We can not solve the problems by using mathematical tools generally in the social life since in mathematics the concepts are precise and not subjective. Some theories were developed to eliminate this lack of vagueness such as fuzzy set theory [1], rough set theory [2], and soft set theory [3].

The fuzzy set theory initiated by Zadeh [1] in 1965 provides a useful framework for modelling and manipulating vague concepts. The fuzzy set theory is based on fuzzy membership function. Fuzzy membership function determines the belongness of an element to a set to a degree. Since being established, this theory has been actively studied by both mathematicians and computer scientists.

The rough set theory [2] proposed by Pawlak in 1982 is an extension of set theory for the study of intelligent systems characterized by insufficient and incomplete information. The classical rough set theory is based on equivalence relations, but it was extended to covering based rough sets [4, 5]. It is well known that the topology and rough set theory have been applied in many science and engineering areas such as chemistry, biology, crystal, image processing, knowledge acquisition, pattern recognition, engineering control, and biomedicine.

In 1999, Molodtsov [3] proposed the concept of a soft set, which can be seen as a new mathematical approach to vagueness. The absence of any restrictions on the approximate description in soft set theory makes this theory very convenient and easily applicable in practice. Maji et al. [6] carried out Molodtsov's idea by introducing several operations in soft set theory. Ali et al. [7] introduced some operations over soft sets. After that, Ge and Yang [8] further investigated these operational rules in [6, 7] and obtained some interesting results including some different viewpoints with [7]. Ge et al. [9] established some relations between topology and soft set theory. Aktaş and Çağman [10] compared soft sets to the related concepts of fuzzy sets and rough sets, providing examples to clarify their differences.

Feng et al. [11] investigated the concept of soft rough set in 2010 which is a combination of soft and rough sets. In [11, 12], basic properties of soft rough approximations were presented and supported by some illustrative examples. In fact, a soft set instead of an equivalence relation was used to granulate the universe of discourse.

In this paper, we investigate the concept of soft covering based rough set which is a combination of covering soft set and rough set. We establish a soft covering approximation space. We supply an example to show that the new type of the soft rough set which is based on covering soft set is more accurate than soft rough set. On the other hand, we find that a new type of the soft covering upper approximation operator is smaller than soft upper approximation operator. Feng [13] gave an application of soft rough approximations in multicriteria group decision making problems and his method enables us to select the optimal object in more reliable manner. In this work, we use soft covering approximations at Feng's method and we present an example in medicine which aims to obtain the optimal choice for applying biopsy to the patients with prostate cancer risk.

Prostate cancer is the second most common cause of cancer death among men in most industrialized countries. It depends on various factors as family's cancer history, age, ethnic background, and the level of prostate specific antigen (PSA) in the blood. Since PSA is a substance produced by the prostate, it is very important factor to an initial diagnosis for patients [14–16]. As known, when the prostate cancer can be diagnosed earlier, the patient can be completely treated. The definitive diagnosis of the prostate cancer is possible with prostate biopsy. The results of PSA test, rectal examination, and transrectal findings help the doctor to decide whether biopsy is necessary or not [17–19]. If there is a biopsy for diagnosing, the cancer may spread to the other vital organs [17]. For this reason, the biopsy method is undesirable. In this study, we aim to reduce the number of patients who applied biopsy. Therefore, we give a new method which determines the necessity of biopsy and it gives to user a range of the risk of the cancer. For this process, it is used as laboratory data, prostate specific antigen (PSA), free prostate specific antigen (fPSA), prostate volume (PV), and age of the patient. We observe that this method is more rapid, economical, and without risk than the traditional diagnostic methods.

## 2. Preliminaries

In this section, we introduce the fundamental ideas behind fuzzy sets, rough sets, soft sets, soft rough sets, and fuzzy soft sets. Throughout this paper, the universe *U* is supposed to be a finite nonempty set, *∅* the empty set, and −*X* the complement of *X* in *U*.

**Definition 1 (see [1]).**

Let *U* be a universe set. A fuzzy set *A* in  *U* is a set of ordered pairs:

(1) $$\begin{array}{c} A=\left\{\left(x,\mu_{A}\left(x\right)\right):x\in U\right\}, \end{array}$$

where *μ*_*A*_ : *U* → [0,1] = *I* is a mapping and *μ*_*A*_(*x*) (or *A*(*x*)) states the grade of belongness of *x* in *A*. The family of all fuzzy sets in *U* is denoted by *I* ^*U*^.

**Definition 2 (see [2]).**

Let *U* be finite set and *R* an equivalence relation on *U*. Then the pair (*U*, *R*) is called a Pawlak approximation space.

*R* generates a partition *U*/*R* = {*Y*_1_, *Y*_2_,…, *Y*_*m*_} on *U*, where *Y*_1_, *Y*_2_,…, *Y*_*m*_ are the equivalence classes generated by the equivalence relation *R*. In the rough set theory, these are also called elementary sets of *R*.

For any *X*⊆*U*, we can describe *X* by the elementary sets of *R* and two sets

(2) R_(X)=∪{Yi∈U/R:Yi⊆X},R¯(X)=∪{Yi∈U/R:Yi∩X≠∅}

which are called the lower and upper approximation of *X*, respectively. In addition,

(3) $$\begin{array}{c} \text{Pos}\left(X\right)=\underline{R}\left(X\right),\\ \text{Neg}\left(X\right)=U-\bar{R}\left(X\right),\\ \text{Bnd}\left(X\right)=\bar{R}\left(X\right)-\underline{R}\left(X\right) \end{array}$$

are called the positive, negative, and boundary regions of *X*, respectively. Now, we are ready to give the definition of rough sets.

**Definition 3 (see [2]).**

If Bnd(*X*) ≠ *∅*, that is, $\bar{R}\left(X\right)\neq \underline{R}\left(X\right)$, *X* is said to be rough (or inexact); in the opposite case, that is, if the boundary region of *X* is empty, that is, $\bar{R}\left(X\right)=\underline{R}\left(X\right)$, then *X* is called definable (or crisp).

**Proposition 4 (see [2]).**

Let (*U*, *R*) be a Pawlak approximation space and *X*, *Y*⊆*U*. The properties of Pawlak's rough sets are

1. $\underline{R}\left(\emptyset \right)=\emptyset ,\bar{R}\left(\emptyset \right)=\emptyset$,
2. $\underline{R}\left(U\right)=U,\bar{R}\left(U\right)=U$,
3. $\underline{R}\left(X\right)\subseteq X\subseteq \bar{R}\left(X\right)$,
4. $X\subseteq Y\Rightarrow \underline{R}\left(X\right)\subseteq \underline{R}\left(Y\right)$,
5. $X\subseteq Y\Rightarrow \bar{R}\left(X\right)\subseteq \bar{R}\left(Y\right)$,
6. $\underline{R}\left(X\cap Y\right)=\underline{R}\left(X\right)\cap \underline{R}\left(Y\right)$,
7. $\bar{R}\left(X\cup Y\right)=\bar{R}\left(X\right)\cup \bar{R}\left(Y\right)$,
8. $\underline{R}(\underline{R}\left(X\right))=\underline{R}\left(X\right)$,
9. $\bar{R}(\bar{R}\left(X\right))=\bar{R}\left(X\right)$,
10. $\underline{R}\left(-X\right)=-\bar{R}\left(X\right)$,
11. $\bar{R}\left(-X\right)=-\underline{R}\left(X\right)$,
12. $\underline{R}(-\underline{R}\left(X\right))=-\underline{R}\left(X\right)$,
13. $\bar{R}(-\bar{R}\left(X\right))=-\bar{R}\left(X\right)$,
14. ∀*K* ∈ *U*/*R*, $\underline{R}\left(K\right)=K$,
15. ∀*K* ∈ *U*/*R*, $\bar{R}\left(K\right)=K$.

Let *U* be an initial universe set and let *E* be the set of all possible parameters with respect to *U*. Parameters are often attributes, characteristics, or properties of the objects in *U*. Let *P*(*U*) denote the power set of *U*. Then a soft set over *U* is defined as follows.

**Definition 5 (see [3]).**

A pair *G* = (*F*, *A*) is called a soft set over *U* where *A*⊆*E* and *F* : *A* → *P*(*U*) is a set valued mapping.

In other words, a soft set over *U* is a parameterized family of subsets of the universe *U*. For ∀*ɛ* ∈ *A*, *F*(*ɛ*) may be considered the set of *ɛ*-approximate elements of the soft set *G* = (*F*, *A*). It is worth noting that *F*(*ɛ*) may be arbitrary. Some of them may be empty and some may have nonempty intersection [3].

**Example 6 .**

Miss Zeynep and Mr. Ahmet are going to marry and they want to hire a wedding room. The soft set (*F*, *E*) describes the “capacity of the wedding room.” Let *U* = {*u*_1_, *u*_2_, *u*_3_, *u*_4_, *u*_5_, *u*_6_} be the wedding rooms under consideration and *E* = {*e*_1_ = big, *e*_2_ = central, *e*_3_ = cheap, *e*_4_ = quality, *e*_5_ = elegant} the parameter set; *F*(*e*_1_) = {*u*_2_, *u*_4_}, *F*(*e*_2_) = {*u*_1_, *u*_3_, *u*_4_}, *F*(*e*_3_) = *∅*, *F*(*e*_4_) = {*u*_1_, *u*_3_, *u*_5_}, *F*(*e*_5_) = {*u*_1_, *u*_6_}. The soft set (*F*, *E*) is as follows: (*F*, *E*) = {*e*_1_ = {*u*_2_, *u*_4_}, *e*_2_ = {*u*_1_, *u*_3_, *u*_4_}, *e*_3_ = *∅*, *e*_4_ = {*u*_1_, *u*_3_, *u*_5_}, *e*_5_ = {*u*_1_, *u*_6_}} (see Table 1).

Although rough sets and soft sets are two different mathematical tools for modelling vagueness, there are some interesting connections between them.

**Theorem 7 (see [10]).**

Every rough set may be considered a soft set.

As pointed out by several researchers, information systems and soft sets are closely related [20, 21]. Given a soft set *G* = (*F*, *A*) over a universe *U*. if *U* and *A* are both nonempty finite sets, then *G* = (*F*, *A*) could induce an information system in a natural way. In fact, for any attribute *a* ∈ *A*, one can define a function *a* : *U* → *V*_*a*_ = {0,1} by

(4) $$\begin{array}{c} a\left(x\right)=\{\begin{array}{cc} 1, & \text{if}\;x\in F\left(a\right),\\ 0, & \text{otherwise}. \end{array} \end{array}$$

Therefore, every soft set may be considered an information system. This justifies the tabular representation of soft sets used widely in the literature. Conversely, it is worth noting that a soft set can also be applied to express an information system. Let *ρ* = (*U*, *A*) be an information system. Taking

(5) $$\begin{array}{c} B=\underset{a\in A}{\cup }\left\{a\right\}\times V_{a} \end{array}$$

as the parameter set, then a soft set (*F*, *B*) can be defined by setting

(6) $$\begin{array}{c} F\left(a,v\right)=\left\{x\in U:a\left(x\right)=v\right\}, \end{array}$$

where *a* ∈ *A* and *v* ∈ *V*_*a*_.

Maji et al. [22] defined the following hybrid model fuzzy soft sets, combining soft sets with fuzzy sets.

**Definition 8 (see [22]).**

Let *A*⊆*E*. (*f*_*A*_, *E*) is defined to be a fuzzy soft set on (*U*, *E*) if *f*_*A*_ : *E* → *I* ^*U*^ is mapping defined by *f*_*A*_(*e*) = *μ*_*f*_*A*__^*e*^, where $\mu_{f_{A}}^{e}=\bar{O}$ if *e* ∈ *E* − *A* and $\mu_{f_{A}}^{e}\neq \bar{O}$ if *e* ∈ *A*, where $\bar{O}(u)=0$ for each *u* ∈ *U*.

**Example 9 (see [23]).**

Miss* X* and Mr.* Y* are going to marry and they want to hire a wedding room. The fuzzy soft set (*f*_*A*_, *E*) describes the “capacity of the wedding room.” Let *U* = {*a*, *b*, *c*, *d*, *e*} be the wedding rooms under consideration, *E* = {big = *e*_1_, central = *e*_2_, cheap = *e*_3_, expensive = *e*_4_, elegant = *e*_5_, quality = *e*_6_, goodserving = *e*_7_} the parameter set, and *A* = {*e*_2_, *e*_5_, *e*_6_} a subset of *E*. Consider (*f*_*A*_, *E*) = {*e*_2_ = {*a*_0.3_, *b*_0.5_, *c*_0.9_, *d*_0.8_, *e*_0.6_}, *e*_5_ = {*a*_0.8_, *b*_0.6_, *c*_0.2_, *d*_0.1_, *e*_0.5_}, *e*_6_ = {*a*_0.7_, *b*_0.5_, *c*_0.3_, *d*_0.2_, *e*_0.4_}} which is a fuzzy soft set on (*U*, *E*).

Feng et al. [11] investigated the concept of soft rough set in 2010 which is a combination of soft and rough sets. A soft set instead of an equivalence relation was used to granulate the universe of discourse. The result was deviation of Pawlak approximation space called a soft approximation space [11]. For more details on this topic, we refer the interested reader to [11, 12]. All proofs can be found there.

**Definition 10 (see [11]).**

Let *G* = (*F*, *A*) be a soft set over *U*. Then the pair *S* = (*U*, *G*) is called a soft approximation space. Based on the approximation space *S*, we define the following two operations:

(7) $$\begin{array}{c} {\underline{\text{apr}}}_{S}\left(X\right)=\left\{u\in U:\exists a\in A,\left[u\in F\left(a\right)\subseteq X\right]\right\},\\ {\bar{\text{apr}}}^{S}\left(X\right)=\left\{u\in U:\exists a\in A,\left[u\in F\left(a\right),F\left(a\right)\cap X\neq \emptyset \right]\right\}, \end{array}$$

assigning to every subset *X*⊆*U* two sets ${\underline{\text{apr}}}_{S}\left(X\right)$ and ${\bar{\text{apr}}}^{S}\left(X\right)$, which are called the soft *S*-lower approximation and the soft *S*-upper approximation of *X*, respectively. In general, we refer to ${\underline{\text{apr}}}_{S}\left(X\right)$ and ${\bar{\text{apr}}}^{S}\left(X\right)$ as soft rough approximations of *X* with respect to *S*.

In addition,

(8) $$\begin{array}{c} \text{Po}{\text{s}}_{S}\left(X\right)={\underline{\text{apr}}}_{S}\left(X\right),\\ \text{Ne}{\text{g}}_{S}\left(X\right)=U-{\bar{\text{apr}}}^{S}\left(X\right),\\ \text{Bn}{\text{d}}_{S}\left(X\right)={\bar{\text{apr}}}^{S}\left(X\right)-{\underline{\text{apr}}}_{S}\left(X\right), \end{array}$$

are called the soft *S*-positive, negative, and boundary regions of *X*, respectively. If ${\bar{\text{apr}}}^{S}\left(X\right)={\underline{\text{apr}}}_{S}\left(X\right)$, *X* is said to be soft *S*-definable; otherwise, *X* is called a soft *S*-rough set.

**Proposition 11 (see [11, 12]).**

Let *G* = (*F*, *A*) be a soft set over *U* and *S* = (*U*, *G*) a soft approximation space. Then we have

(9) $$\begin{array}{c} {\underline{apr}}_{S}\left(X\right)=\underset{a\in A}{\cup }\left\{F\left(a\right):F\left(a\right)\subseteq X\right\},\\ {\bar{apr}}^{S}\left(X\right)=\underset{a\in A}{\cup }\left\{F\left(a\right):F\left(a\right)\cap X\neq \emptyset \right\} \end{array}$$

for all *X*⊆*U*.

**Theorem 12 (see [11, 12]).**

Let *G* = (*F*, *A*) be a soft set over *U* and *S* = (*U*, *G*) a soft approximation space and *X*, *Y*⊆*U*. Then the soft *S*-lower and upper approximations have the following properties:

1. ${\underline{apr}}_{S}\left(\emptyset \right)={\bar{apr}}^{S}\left(\emptyset \right)=\emptyset$,
2. ${\underline{apr}}_{S}\left(U\right)={\bar{apr}}^{S}\left(U\right)=\cup_{a\in A}F\left(a\right)$,
3. ${\underline{apr}}_{S}\left(X\right)\subseteq X$,
4. ${\underline{apr}}_{S}\left(X\right)\subseteq {\bar{apr}}^{S}\left(X\right)$,
5. $X\subseteq Y\Rightarrow {\underline{apr}}_{S}\left(X\right)\subseteq {\underline{apr}}_{S}\left(Y\right)$,
6. $X\subseteq Y\Rightarrow {\bar{apr}}^{S}\left(X\right)\subseteq {\bar{apr}}^{S}\left(Y\right)$,
7. ${\underline{apr}}_{S}\left(X\cap Y\right)\subseteq {\underline{apr}}_{S}\left(X\right)\cap {\underline{apr}}_{S}\left(Y\right)$,
8. ${\underline{apr}}_{S}\left(X\cup Y\right)⊇{\underline{apr}}_{S}\left(X\right)\cup {\underline{apr}}_{S}\left(Y\right)$,
9. ${\bar{apr}}^{S}\left(X\cup Y\right)={\bar{apr}}^{S}\left(X\right)\cup {\bar{apr}}^{S}\left(Y\right)$,
10. ${\bar{apr}}^{S}\left(X\cap Y\right)\subseteq {\bar{apr}}^{S}\left(X\right)\cap {\bar{apr}}^{S}\left(Y\right)$.

**Definition 13 (see [12]).**

A soft set *G* = (*F*, *A*) over *U* is called a full soft set if ∪_*a*∈*A*_ *F*(*a*) = *U*.

**Theorem 14 (see [12]).**

Let *G* = (*F*, *A*) be a soft set over *U* and *S* = (*U*, *G*) a soft approximation space. Then the following conditions are equivalent:

1. *S* is a full soft set
2. ${\underline{apr}}_{S}\left(U\right)=U$,
3. ${\bar{apr}}^{S}\left(U\right)=U$,
4. $X\subseteq {\bar{apr}}^{S}\left(X\right)$ for all *X*⊆*U*,
5. ${\underline{apr}}_{S}\left(\left\{u\right\}\right)\neq \emptyset$ for all *u* ∈ *U*.

To show the relationship between soft rough sets and Pawlak's rough sets, we first observe that soft sets and binary relations are closely related [11, 12].

**Theorem 15 (see [11, 12]).**

Let *G* = (*F*, *A*) be a soft set over *U*. Then *G* = (*F*, *A*) induces a binary relation *R*_*G*_⊆*A* × *U*, which is defined by

(10) $$\begin{array}{c} \left(x,y\right)\in R_{G}⟺y\in F\left(x\right) \end{array}$$

for all *x* ∈ *A*, *y* ∈ *U*.

Conversely, assume that *R* is a binary relation from *A* to *U*. Define a set valued mapping *F*_*R*_ : *A* → *P*(*U*) by

(11) $$\begin{array}{c} F_{R}\left(x\right)=\left\{y\in U:\left(x,y\right)\in R\right\}, \end{array}$$

where all *x* ∈ *A*. Then *G*_*R*_ = (*F*_*R*_, *A*) is a soft set over *U*. Moreover, it is seen that *G*_*R*_*G*__ = *G* and *R*_*G*_*R*__ = *R*.

The following results show that Pawlak's rough set model can be viewed as a special case of soft rough sets [12].

**Theorem 16 (see [12]).**

Let *R* be an equivalence relation on *U*, *G*_*R*_ = (*F*_*R*_, *U*) the canonical soft set of *R*, and *S* = (*U*, *G*_*R*_) a soft approximation space. Then, for all *X*⊆*U*,

(12) $$\begin{array}{c} \underline{R}\left(X\right)={\underline{apr}}_{S}\left(X\right),\;\;\bar{R}\left(X\right)={\bar{apr}}^{S}\left(X\right), \end{array}$$

where $\underline{R}\left(X\right)$ and $\bar{R}\left(X\right)$ are the Pawlak rough approximations of *X*. Thus, in this case, *X*⊆*U* is a (Pawlak) rough set if and only if *X* is a soft *S*-rough set.

**Theorem 17 (see [12]).**

Let *G* = (*F*, *A*) be a partition soft set over *U* and *S* = (*U*, *G*) a soft approximation space. Define an equivalence relation *R* on *U* by

(13) $$\begin{array}{c} \left(x,y\right)\in R⟺\exists a\in A,\;\;\left\{x,y\right\}\subseteq F\left(a\right) \end{array}$$

for all *x*, *y* ∈ *U*. Then, for all *X*⊆*U*,

(14) $$\begin{array}{c} \underline{R}\left(X\right)={\underline{apr}}_{S}\left(X\right),\;\;\bar{R}\left(X\right)={\bar{apr}}^{S}\left(X\right). \end{array}$$

## 3. Soft Covering Based Rough Sets

In this section, we use a special kind of soft set with rough set and establish a soft covering approximation space and present its basic properties.

**Definition 18 (see [11]).**

A full soft set *G* = (*F*, *A*) over *U* is called a covering soft set if *F*(*a*) ≠ *∅*, ∀*a* ∈ *A*.

We indicate a covering soft set with *C*_*G*_.

**Definition 19 .**

Let *G* = (*F*, *A*) be a covering soft set over *U*. Then the pair *S* = (*U*, *C*_*G*_) is called a soft covering approximation space.

**Definition 20 .**

Let *S* = (*U*, *C*_*G*_) be a soft covering approximation space and *x* ∈ *U*. Then the soft minimal description of *x* is defined as follows: *Md*_*S*_(*x*) = {*F*(*a*) : *a* ∈ *A*∧*x* ∈ *F*(*a*)∧(∀*e* ∈ *A*∧*x* ∈ *F*(*e*)⊆*F*(*a*)⇒*F*(*a*) = *F*(*e*))}.

In order to describe an object, we need only the essential characteristics related to this object, not all characteristics for this object. That is the purpose of minimal description concept.

**Definition 21 .**

Let *S* = (*U*, *C*_*G*_) be a soft covering approximation space. For a set *X*⊆*U*, soft covering lower and upper approximations are, respectively, defined as

(15) S_∗(X)=∪a∈A{F(a):F(a)⊆X},S¯∗(X)=∪{MdS(x):x∈X}.

In addition,

(16) $$\begin{array}{c} \text{Po}{\text{s}}_{S}\left(X\right)={\underline{S}}_{*}\left(X\right),\\ \text{Ne}{\text{g}}_{S}\left(X\right)=U-{\bar{S}}^{*}\left(X\right),\\ \text{Bn}{\text{d}}_{S}\left(X\right)={\bar{S}}^{*}\left(X\right)-{\underline{S}}_{*}\left(X\right) \end{array}$$

are called the soft covering positive, negative, and boundary regions of *X*, respectively.

**Definition 22 .**

Let *S* = (*U*, *C*_*G*_) be a soft covering approximation space. A subset *X*⊆*U* is called soft covering based definable if ${\bar{S}}^{*}\left(X\right)={\underline{S}}_{*}\left(X\right)$; in the opposite case, that is, if ${\bar{S}}^{*}\left(X\right)\neq {\underline{S}}_{*}\left(X\right)$, *X* is said to be soft covering based rough set.

**Example 23 .**

Let *U* = {*u*_1_, *u*_2_, *u*_3_, *u*_4_, *u*_5_, *u*_6_} be universe and *G* = (*F*, *E*) a covering soft set over *U*, where *E* = {*e*_1_, *e*_2_, *e*_3_, *e*_4_, *e*_5_, *e*_6_}, *F*(*e*_1_) = {*u*_1_, *u*_2_}, *F*(*e*_2_) = {*u*_1_, *u*_2_, *u*_3_, *u*_4_}, *F*(*e*_3_) = {*u*_3_, *u*_4_}, *F*(*e*_4_) = {*u*_3_, *u*_4_, *u*_5_, *u*_6_}, *F*(*e*_5_) = {*u*_1_, *u*_2_, *u*_5_, *u*_6_}, and *F*(*e*_6_) = {*u*_3_, *u*_5_, *u*_6_}. Then *S* = (*U*, *C*_*G*_) is a soft covering approximation space. For *X*_1_ = {*u*_1_, *u*_2_}⊆*U*, we have

(17) S_∗(X1)=∪e∈E{F(e):F(e)⊆X1}={u1,u2},S¯∗(X1)=∪{MdS(x):x∈X1}={u1,u2}.

Thus, ${\underline{S}}_{*}\left(X_{1}\right)={\bar{S}}^{*}\left(X_{1}\right)$ and *X*_1_ is a soft covering based definable set. For *X*_2_ = {*u*_1_, *u*_2_, *u*_4_}⊆*U*, we have

(18) S_∗(X2)=∪e∈E{F(e):F(e)⊆X2}={u1,u2}S¯∗(X2)=∪{MdS(x):x∈X2}={u1,u2,u3,u4}.

Thus, ${\underline{S}}_{*}\left(X_{2}\right)\neq {\bar{S}}^{*}\left(X_{2}\right)$ and *X*_2_ is a soft covering based rough set.

A soft rough set is based on a soft set in a soft approximation space, whereas a soft covering based rough set is based on a covering soft set in a soft covering approximation space. We can call the soft rough set which is given in [11] as the first type of soft covering based rough set in a soft covering approximation space. From the definitions of two types of soft covering lower approximation operations, it is easy to see that the new soft covering lower approximation is the same as that in the first type of soft covering based rough set model. Therefore, we can give the following results.

**Corollary 24 .**

Let *G* = (*F*, *A*) be a covering soft set over *U*, *S* = (*U*, *C*_*G*_) a soft covering approximation space and *X*, *Y*⊆*U*. Then the soft covering lower and upper approximations have the following properties:

1. ${\underline{apr}}_{S}\left(\emptyset \right)={\bar{apr}}^{S}\left(\emptyset \right)=\emptyset$,
2. ${\underline{apr}}_{S}\left(U\right)={\bar{apr}}^{S}\left(U\right)=\cup_{a\in A}F\left(a\right)=U$,
3. ${\underline{apr}}_{S}\left(X\right)\subseteq X$,
4. ${\underline{apr}}_{S}\left(X\right)\subseteq {\bar{apr}}^{S}\left(X\right)$,
5. $X\subseteq Y\Rightarrow {\underline{apr}}_{S}\left(X\right)\subseteq {\underline{apr}}_{S}\left(Y\right)$,
6. $X\subseteq Y\Rightarrow {\bar{apr}}^{S}\left(X\right)\subseteq {\bar{apr}}^{S}\left(Y\right)$,
7. ${\underline{apr}}_{S}\left(X\cap Y\right)\subseteq {\underline{apr}}_{S}\left(X\right)\cap {\underline{apr}}_{S}\left(Y\right)$,
8. ${\underline{apr}}_{S}\left(X\cup Y\right)⊇{\underline{apr}}_{S}\left(X\right)\cup {\underline{apr}}_{S}\left(Y\right)$,
9. ${\bar{apr}}^{S}\left(X\cup Y\right)={\bar{apr}}^{S}\left(X\right)\cup {\bar{apr}}^{S}\left(Y\right)$,
10. ${\bar{apr}}^{S}\left(X\cap Y\right)\subseteq {\bar{apr}}^{S}\left(X\right)\cap {\bar{apr}}^{S}\left(Y\right)$,
11. $X\subseteq {\bar{apr}}^{S}\left(X\right)$ for all *X*⊆*U*,
12. ${\underline{apr}}_{S}\left(\left\{u\right\}\right)\neq \emptyset$ for all *u* ∈ *U*.

**Proof**

The proof is a direct consequence of Theorems 12 and 14.

From the definitions of two types of soft covering upper approximation operations, we have ${\bar{S}}^{*}\left(X\right)\subseteq {\bar{\text{apr}}}^{S}\left(X\right)$ for a set *X*⊆*U*. But ${\bar{\text{apr}}}^{S}\left(X\right)\subseteq {\bar{S}}^{*}\left(X\right)$ is not true in general as shown in the following example.

**Example 25 .**

From Example 23, for *X* = {*u*_2_, *u*_4_}⊆*U*, we have

(19) S¯∗(X)=∪{MdS(x):x∈X}={u1,u2,u3,u4},apr¯S(X)=∪a∈A{F(a):F(a)∩X≠∅}=U.

Thus, ${\bar{\text{apr}}}^{S}\left(X\right)⊊{\bar{S}}^{*}\left(X\right)$.

Now, we investigate some properties of the new type of soft covering upper approximation.

**Theorem 26 .**

Let *G* = (*F*, *A*) be a covering soft set over *U* and *S* = (*U*, *C*_*G*_) a soft covering approximation space and *X*, *Y*⊆*U*. Then the soft covering upper approximation has the following properties:

1. ${\bar{S}}^{*}\left(U\right)=U$,
2. ${\bar{S}}^{*}\left(\emptyset \right)=\emptyset$,
3. $X\subseteq {\bar{S}}^{*}\left(X\right)$,
4. ${\bar{S}}^{*}\left(X\cup Y\right)={\bar{S}}^{*}\left(X\right)\cup {\bar{S}}^{*}\left(Y\right)$,
5. $X\subseteq Y\Rightarrow {\bar{S}}^{*}\left(X\right)\subseteq {\bar{S}}^{*}\left(Y\right)$

**Proof**

From Definition 21, we can easily prove properties 1, 2, and 3.

(4) From Definition 21, we have ${\bar{S}}^{*}\left(X\right)=\cup \{Md_{S}(x):x\in X\}$ and ${\bar{S}}^{*}\left(Y\right)=\cup \{Md_{S}(x):x\in Y\}$. So ${\bar{S}}^{*}\left(X\right)\cup {\bar{S}}^{*}\left(Y\right)=$ (∪{*Md*_*S*_(*x*) : *x* ∈ *X*})∪ (∪{*Md*_*S*_(*x*) : *x* ∈ *Y*})  = ∪{*Md*_*S*_(*x*) : *x* ∈ *X* ∪ *Y*} =  ${\bar{S}}^{*}\left(X\cup Y\right)$.

(5) By the definition of ${\bar{S}}^{*}\left(X\right)=\cup \{Md_{S}(x):x\in X\},x_{1},x_{2},\ldots ,x_{m}\in X$, and *F*(*a*_1_), *F*(*a*_2_),…, *F*(*a*_*m*_) ∈ *G* = (*F*, *A*) such that *F*(*a*_*i*_) ∈ *Md*_*S*_(*x*_*i*_), 1 ≤ *i* ≤ *m*,  ${\bar{S}}^{*}\left(X\right)$ is expressed as ${\bar{S}}^{*}\left(X\right)=F\left(a_{1}\right)\cup F\left(a_{2}\right)\cup \cdots \cup F\left(a_{m}\right)$. It is obvious that $F\left(a_{i}\right)\subseteq {\bar{S}}^{*}\left(X\right).$ Since *X*⊆*Y* and *x*_1_, *x*_2_,…, *x*_*m*_ ∈ *X*, we obtain *x*_*i*_ ∈ *Y*. For 1 ≤ *i* ≤ *m*, $F\left(a_{i}\right)\subseteq {\bar{S}}^{*}\left(Y\right)$. Therefore, ${\bar{S}}^{*}\left(X\right)\subseteq {\bar{S}}^{*}\left(Y\right)$.

**Theorem 27 .**

Let *G* = (*F*, *A*) be a covering soft set over *U* and *S* = (*U*, *C*_*G*_) a soft covering approximation space and *X*, *Y*⊆*U*. Then the soft covering lower and upper approximations do not have the following properties:

1. ${\underline{S}}_{*}\left(X\cap Y\right)={\underline{S}}_{*}\left(X\right)\cap {\underline{S}}_{*}\left(Y\right)$,
2. ${\underline{S}}_{*}\left(-{\underline{S}}_{*}\left(X\right)\right)=-{\underline{S}}_{*}\left(X\right)$,
3. ${\bar{S}}^{*}(-{\bar{S}}^{*}\left(X\right))=-{\bar{S}}^{*}\left(X\right)$,
4. ${\bar{S}}^{*}({\bar{S}}^{*}\left(X\right))={\bar{S}}^{*}\left(X\right)$,
5. ${\underline{S}}_{*}\left(X\right)=-{\bar{S}}^{*}\left(-X\right)$,
6. ${\bar{S}}^{*}\left(X\right)=-{\underline{S}}_{*}\left(-X\right)$,
7. ∀*a* ∈ *A*, ${\bar{S}}^{*}\left(F\left(a\right)\right)=F\left(a\right)$.

The following examples show that the equalities mentioned above do not hold.

**Example 28 .**

Let *U* = {*u*_1_, *u*_2_, *u*_3_, *u*_4_, *u*_5_} be universe and *G* = (*F*, *E*) a covering soft set over *U*, where *E* = {*e*_1_, *e*_2_, *e*_3_, *e*_4_}, *F*(*e*_1_) = {*u*_1_, *u*_2_}, *F*(*e*_2_) = {*u*_2_, *u*_3_}, *F*(*e*_3_) = {*u*_3_, *u*_4_}, and *F*(*e*_4_) = {*u*_4_, *u*_5_}. Then *S* = (*U*, *C*_*G*_) is a soft covering approximation space.

1. Suppose that *X* = {*u*_1_, *u*_2_}⊆*U*, $Y=\{u_{2},u_{3},u_{4}\}\subseteq U.\;{\underline{S}}_{*}\left(X\right)=\{u_{1},u_{2}\}$, $\;{\underline{S}}_{*}\left(Y\right)=\left\{u_{2},u_{3},u_{4}\right\}$, ${\underline{S}}_{*}\left(X\right)\cap {\underline{S}}_{*}\left(Y\right)=\{u_{2}\}$, and ${\underline{S}}_{*}\left(X\cap Y\right)=\emptyset$. This shows that ${\underline{S}}_{*}\left(X\cap Y\right)\neq {\underline{S}}_{*}\left(X\right)\cap {\underline{S}}_{*}\left(Y\right)$.
2. Suppose that $X=\{u_{1},u_{2},u_{4},u_{5}\}\subseteq U.\;{\underline{S}}_{*}\left(X\right)=\{u_{1},u_{2},u_{4},u_{5}\}$, $-{\underline{S}}_{*}\left(X\right)=\{u_{3}\},\;{\underline{S}}_{*}\left(-{\underline{S}}_{*}\left(X\right)\right)=\emptyset$. This shows that ${\underline{S}}_{*}\left(-{\underline{S}}_{*}\left(X\right)\right)\neq -{\underline{S}}_{*}\left(X\right)$.
3. Suppose that $X=\{u_{1}\}\subseteq U.\;{\bar{S}}^{*}\left(X\right)=\{u_{1},u_{2}\}$,  $-{\bar{S}}^{*}\left(X\right)=\{u_{3},u_{4},u_{5}\}$, ${\bar{S}}^{*}(-{\bar{S}}^{*}\left(X\right))=\{u_{2},u_{3},u_{4},u_{5}\}$. This shows that ${\bar{S}}^{*}(-{\bar{S}}^{*}\left(X\right))\neq -{\bar{S}}^{*}\left(X\right)$.
4. Suppose that $X=\{u_{1}\}\subseteq U.\;{\bar{S}}^{*}\left(X\right)=\{u_{1},u_{2}\}$, ${\bar{S}}^{*}({\bar{S}}^{*}\left(X\right))=\{u_{1},u_{2},u_{3}\}$. This shows that ${\bar{S}}^{*}({\bar{S}}^{*}\left(X\right))\neq {\bar{S}}^{*}\left(X\right)$.
5. Suppose that $X=\{u_{1},u_{2},u_{4},u_{5}\}\subseteq U.\;{\underline{S}}_{*}\left(X\right)=\{u_{1},u_{2},u_{4},u_{5}\}$, ${\bar{S}}^{*}\left(-X\right)=\{u_{2},u_{3},u_{4}\}$,  $-{\bar{S}}^{*}\left(-X\right)=\{u_{1},u_{5}\}$. This shows that ${\underline{S}}_{*}\left(X\right)\neq -{\bar{S}}^{*}\left(-X\right)$.
6. Suppose that $X=\{u_{1}\}\subseteq U.\;{\bar{S}}^{*}\left(X\right)=\{u_{1},u_{2}\}$, ${\underline{S}}_{*}\left(-X\right)=\{u_{2},u_{3},u_{4},u_{5}\}$, and $-{\underline{S}}_{*}\left(-X\right)=\{u_{1}\}$. This shows that ${\bar{S}}^{*}\left(X\right)\neq -{\underline{S}}_{*}\left(-X\right)$.
7. *F*(*e*_1_) = {*u*_1_, *u*_2_}, ${\bar{S}}^{*}\left(F\left(e_{1}\right)\right)=\{u_{1},u_{2},u_{3}\}$. This shows that *e*_1_ ∈ *E*, ${\bar{S}}^{*}\left(F\left(e_{1}\right)\right)\neq F\left(e_{1}\right)$.

## 4. Multicriteria Group Decision Making

Feng [13] applied soft rough sets to multicriteria group decision making problem. The soft rough set based decision making method in [13] can be summarized as follows.

**Step 1 .**

Input the original description soft set *G* = (*F*, *A*).

**Step 2 .**

Construct the evaluation soft set *G*_1_ = (*V*, *T*) using the primary evaluation results of the expert group *T*.

**Step 3 .**

Compute soft rough approximations and then obtain the soft sets $G_{1_{-}}=(\underline{V},T)$ and $G_{1}^{-}=(\bar{V},T)$.

**Step 4 .**

Compute the corresponding fuzzy sets *μ*_*G*_1__, *μ*_*G*_1_−___, and *μ*_*G*_1_^−^_ of the soft sets *G*_1_ = (*V*, *T*), $G_{1_{-}}=(\underline{V},T)$, and $G_{1}^{-}=(\bar{V},T)$.

**Step 5 .**

Construct the fuzzy soft set *G*_*F*_ = (*α*, *C*) using the fuzzy soft sets *μ*_*G*_1_−___, *μ*_*G*_1__, and *μ*_*G*_1_^−^_.

**Step 6 .**

Input the weighting vector *R* and compute the weighted evaluation values *w*(*u*_*k*_) of each alternative *u*_*k*_ ∈ *U*. Then rank all the alternatives according to their weighted evaluation values; one can select any of the objects with the largest weighted evaluation value as the most preferred alternative.

We use this method to help doctors in diagnosing the prostate cancer risk. In our work, we use soft covering approximations instead of soft rough approximations in Step 3. We may expect to gain much more useful information with the help of soft covering approximations.

## 5. An Application of Multicriteria Group Decision Making by New Type of Soft Covering Approximation Operators

Feng [13] gave an application of soft rough approximations in multicriteria group decision making problems and his method enables us to select the optimal object in more reliable manner. In this work, we used soft covering approximations at Feng's method and aim to obtain the optimal choice for applying biopsy to the patients with prostate cancer risk by using the PSA, fPSA, PV, and age data of patients. We determine the risk of prostate cancer. Our aim is to help the doctor to determine whether the patient needs biopsy or not.

We choose 78 patients from Selçuk University Medicine Faculty with prostate complaint as the data (see Table 2).

**Step 1 .**

Let *U* = {*u*_*k*_ : *u*_1_ = 1, *u*_2_ = 2,…, *u*_78_ = 78, *k* = 1,…, 78} be the universe and *A* = {PSA, fPSA, PV, Age} the parameter set. Now we obtain parameterized subsets of the universe. The patients whose PSA in blood is 50 and higher than 50, fPSA is 12 and bigger than 12, PV is 20 and bigger than 20, and age is 54 and older than 54 are chosen with doctor's suggestion. We generate the soft set *G* = (*F*, *A*) which is based on PSA, fPSA, PV, and age values of patients over *U* (see Table 3). Since *G* = (*F*, *A*) is a covering soft set, *S* = (*U*, *C*_*G*_) is the soft covering approximation space. Consider the following:

(20) f(PSA)={1,4,6,7,9,11,13,15,16,18,19,20,22,23,25,26,28,29,31,33,34,36,37,39,40,42,43,45,46,47,48,49,52,53,55,56,58,60,62,63,64,66,68,70,71,72,73,74,75,77},f(fPSA)={1,4,7,8,9,10,11,13,15,16,17,18,19,20,22,23,24,25,26,28,29,31,32,33,34,35,36,37,39,40,42,43,45,46,48,49,51,52,53,55,56,58,60,62,63,64,66,68,70,71,72,73,74,75,76,77,78}f(Age)={1,…,29,31,…,56,58,60,…,78},f(PV)=U.

**Step 2 .**

Let *T* = {*T*_*d*_1__, *T*_*d*_2__, *T*_*d*_3__} be the specialist doctors group who evaluate the patients with respect to the parameters PSA, fPSA, PV, and age. Now we generate the soft set *G*_1_ = (*V*, *T*) over *U* by using the first evaluation of the results of specialist doctors group *T*. Each specialist needs to examine all the objects in *U* = {*u*_*k*_ : *u*_1_ = 1, *u*_2_ = 2,…, *u*_78_ = 78, *k* = 1,…, 78} and will be requested to only point out “the optimal alternatives” as his/her evaluation result. Hence, each specialist's primary evaluation results are subsets of 78 patients from Selçuk University Medicine Faculty with prostate complaint as the data. For simplicity, we assume that the evaluations of these specialists in *T* = {*T*_*d*_1__, *T*_*d*_2__, *T*_*d*_3__} are of the same importance:

(21) Xd1=V(Td1)={1,4,6,7,8,9,11,13,15,16,17,18,19,20,22,23,25,26,28,29,31,33,34,36,37,39,40,41,42,43,45,46,47,48,49,51,52,53,55,56,58,60,62,63,64,66,68,70,71,72,73,74,75,77},Xd2=V(Td2)={1,2,3,4,6,7,9,11,13,15,16,18,20,22,23,24,25,26,28,29,31,33,34,36,37,39,40,41,42,43,45,46,47,48,49,51,52,53,55,56,58,60,62,64,66,68,70,72,73,74,75,77,78},Xd3=V(Td3)={1,2,4,6,7,9,11,13,15,16,18,19,20,22,23,25,26,28,29,31,33,34,36,37,39,40,41,42,43,45,46,47,48,49,51,52,53,54,55,56,58,60,62,64,66,67,68,70,72,73,75,76,77,78}.

**Step 3 .**

Now, we show how to use second type of soft covering based rough sets to support this group decision making process. We consider the soft rough approximations of the specialist *T*_*d*_*i*__'s primary evaluation result *X*_*d*_*i*__ with respect to the soft approximation space. Let us choose *S* = (*U*, *C*_*G*_) as the soft covering approximation space. By using the soft covering approximations, we obtain two other soft sets $G_{1_{-}}=(\underline{V},T)$ and $G_{1}^{-}=(\bar{V},T)$ over *U*, where

(22) V_:T⟶P(U), V_(Tdi)=S_∗(Xdi), i=1,2,3,V¯:T⟶P(U), V¯(Tdi)=S¯∗(Xdi), i=1,2,3.

The soft set *G*_1_^−^ can be seen as the evaluation result of the specialist doctor group *T* with low confidence while the soft set *G*_1_−__ represents the evaluation result of the specialist doctor group *T* with high confidence.

Now we obtain the soft covering upper and lower approximations of three specialist doctors first evaluation results to get the soft sets *G*_1_^−^ and *G*_1_−__. Consider

(23) V_(Td1)=S_∗(Xd1)=F(PSA),V_(Td2)=S_∗(Xd2)=∅,V_(Td3)=S_∗(Xd3)=∅,V¯(Td1)=S¯∗(Xd1)=F(PSA)∪F(fPSA)∪F(age)=F(age),V¯(Td2)=S¯∗(Xd2)=F(PSA)∪F(fPSA)∪F(age)=F(age),V¯(Td3)=S¯∗(Xd3)=F(PSA)∪F(fPSA)∪F(age)=F(age).

**Step 4 .**

The results of the specialist three doctors evaluation can be formulized in terms of fuzzy sets. For *X*⊆*U*, the characteristic function of *X* is denoted by *χ*_*X*_. Based on the soft set *G*_1_ = (*V*, *T*), we can define fuzzy set *μ*_*G*_1__ in *U* by

(24) μG1:U⟶[0,1],uk⟶μG1(uk)=13∑i=13χV(Tdi)(uk),

where *V*(*T*_*d*_*i*__) = *X*_*d*_*i*__ and *k* = 1,…, 78; *i* = 1,2, 3.

In a similar way, we can get the fuzzy sets *μ*_*G*_1_−___ and *μ*_*G*_1_^−^_ as follows:

(25) μG1−:U⟶[0,1],uk⟶μG1−(uk)=13∑i=13χV_(Tdi)(uk),μG1−:U⟶[0,1],uk⟶μG1−(uk)=13∑i=13χV¯(Tdi)(uk),

where $\underline{V}(T_{d_{i}})={\underline{S}}_{*}(X_{d_{i}})$, $\bar{V}(T_{d_{i}})={\bar{S}}^{*}(X_{d_{i}})$, and *k* = 1,…, 78; *i* = 1,2, 3.

From *G*_1_−__⊆*G*_1_⊆*G*_1_^−^, it is easy to see that *μ*_*G*_1_−___⊆*μ*_*G*_1__⊆*μ*_*G*_1_^−^_. These fuzzy sets *μ*_*G*_1_−___, *μ*_*G*_1__, *μ*_*G*_1_^−^_ can be interpreted as some vague concepts like “the patients under high risk,” “the patients under middle risk,” and “the patients under low risk,” respectively.

By this way, we obtain the fuzzy sets *μ*_*G*_1__, *μ*_*G*_1_−___, *μ*_*G*_1_^−^_ by the memberships we get above. For example, we obtain these fuzzy sets for the first patient:

(26) $$\begin{array}{c} \mu_{G_{1}}\left(1\right)=1,\;\mu_{G_{1_{-}}}\left(1\right)=\frac{1}{3},\;\mu_{G_{1}^{-}}\left(1\right)=1. \end{array}$$

**Step 5 .**

Let *C* = {*L*, *M*, *H*} be a set of parameters, where *L*, *M*, and *H* denote “under low risk,” “under middle risk,” and “under high risk,” respectively. Now we can define a fuzzy soft set *G*_*F*_ = (*α*, *C*) over *U*, where *α* : *C* → *I* ^*U*^ is given by *α*(*L*) = *μ*_*G*_1_^−^_, *α*(*M*) = *μ*_*G*_1__, and *α*(*H*) = *μ*_*G*_1_−___.

**Step 6 .**

Given a weighting vector *R* = (*r*_*L*_, *r*_*M*_, *r*_*H*_) such that *r*_*L*_ + *r*_*M*_ + *r*_*H*_ = 1,

(27) $$\begin{array}{c} w\left(u_{k}\right)=r_{L}\cdot \alpha \left(L\right)\left(u_{k}\right)+r_{M}\cdot \alpha \left(M\right)\left(u_{k}\right)+r_{H}\cdot \alpha \left(H\right)\left(u_{k}\right) \end{array}$$

is called the weighted evaluation value of the alternative *u*_*k*_ ∈ *U*, *k* = 1,…, 78. Assume that the weighting vector *R* = (0.25, 0.5, 0.25). Finally, we can select the object *u*_*p*_ such that *w*(*u*_*p*_) = max⁡{*w*(*u*_*k*_) : *k* = 1,…, 78} as the patient with the highest cancer risk (see Table 4).

When we rank all the alternatives according to their weighted evaluation values, we can select any of the objects with the largest weighted evaluation value as the highest cancer risk. The results are as follows:

(28) 1≈4≈6≈7≈9≈11≈13≈15≈16≈18≈20≈22≈23≈25≈26≈28≈29≈31≈33≈34≈36≈37≈39≈40≈42≈43≈45≈46≈47≈48≈49≈52≈53≈55≈56≈58≈60≈62≈64≈66≈68≈70≈72≈73≈75≈77=0,83>41≈51=0,75>19≈74=0,67>2≈78=0,58>63≈71=0,5>3≈8≈17≈24≈54≈67≈76=0,42>5≈10≈12≈14≈21≈27≈32≈35≈38≈44≈50≈61≈65≈69=0,25>30≈57≈59=0.

Our results show that 0,83 is the highest value and 46 patients have this value and the patients with the membership 0,83 are potential cancer patients and they are under the highest risk. They need biopsy exactly. Two patients with 0,75 value are also under middle risk and they should be followed up by the doctor. The other patients are under low risk and they do not need the biopsy.

According to data from Selçuk University Medicine Faculty, the biopsy is applied to all 78 patients, but only 44 patients were diagnosed with cancer. That is, 34 patients do not need the biopsy. According to our study, we obtained that the biopsy is necessary only to a group of 46 patients who are under high cancer risk. This group also contains 44 patients who were diagnosed with cancer. Hence, we reduce the number of patients who need biopsy.

## Figures and Tables

**Table 1:** Tabular presentation of the soft set.

*U*	*e*_1_	*e*_2_	*e*_3_	*e*_4_	*e*_5_
*u*_1_	0	1	0	1	1
*u*_2_	1	0	0	0	0
*u*_3_	0	1	0	1	0
*u*_4_	1	1	0	0	0
*u*_5_	0	0	0	1	0
*u*_6_	0	0	0	0	1

**Table 2:** The input PSA, fPSA, PV, and age values of several patients.

*U*	PSA	fPSA	PV	Age
*u*_1_	76	17	30	65
*u*_5_	39	7	48	64
*u*_21_	39	9	52	68
*u*_30_	27	7	28	51
*u*_46_	88	19	37	77
*u*_51_	46	12	62	71
*u*_54_	42	10	59	80
*u*_71_	52	12	35	65
*u*_74_	51	12	78	67
*u*_78_	41	13	79	80

**Table 3:** Tabular presentation of the soft set *G* = (*F*, *A*).

*U*	PSA	fPSA	PV	Age
*u*_1_	1	1	1	1
*u*_5_	0	0	1	1
*u*_21_	0	0	1	1
*u*_30_	0	0	1	0
*u*_46_	1	1	1	1
*u*_51_	0	1	1	1
*u*_54_	0	0	1	1
*u*_71_	1	1	1	1
*u*_74_	1	1	1	1
*u*_78_	0	1	1	1

**Table 4:** Tabular presentation of the fuzzy soft set *G*_*F*_ = (α, *C*) with weighted evaluation value of several patients.

*U*	μ_*G*_1_−___	μ_*G*_1__	μ_*G*_1_^−^_	*w*(*u*_*k*_)
*u*_1_	1l3	1	1	0,83
*u*_5_	0	0	1	0,25
*u*_21_	0	0	1	0,25
*u*_30_	0	0	0	0
*u*_46_	$\frac{1}{3}$	1	1	0,83
*u*_51_	0	1	1	0,75
*u*_54_	0	$\frac{1}{3}$	1	0,42
*u*_71_	$\frac{1}{3}$	$\frac{1}{3}$	1	0,5
*u*_74_	$\frac{1}{3}$	$\frac{2}{3}$	1	0,67
*u*_78_	0	$\frac{2}{3}$	1	0,58
